# Synthesis and Characterization of Novel 2-Amino-Chromene-Nitriles that Target Bcl-2 in Acute Myeloid Leukemia Cell Lines

**DOI:** 10.1371/journal.pone.0107118

**Published:** 2014-09-30

**Authors:** Hosadurga K. Keerthy, Manoj Garg, Chakrabhavi D. Mohan, Vikas Madan, Deepika Kanojia, Rangappa Shobith, Shivananju Nanjundaswamy, Daniel J. Mason, Andreas Bender, Basappa, Kanchugarakoppal S. Rangappa, H. Phillip Koeffler

**Affiliations:** 1 Laboratory of Chemical Biology, Department of Chemistry, Bangalore University, Bangalore, India; 2 Genomic Oncology Programme, Cancer Science Institute of Singapore, National University of Singapore, Singapore, Singapore; 3 Department of Studies in Chemistry, University of Mysore, Manasagangotri, Mysore, India; 4 Interdisciplinary Research Group of Infectious Diseases, Singapore-MIT Alliance for Research & Technology Centre (SMART), Singapore, Singapore; 5 Unilever Centre for Molecular Science Informatics, Department of Chemistry, University of Cambridge, Cambridge, United Kingdom; 6 Division of Hematology and Oncology, Cedar-Sinai Medical Centre, Los Angeles, California, United States of America; University of Parma, Italy

## Abstract

The anti-apoptotic protein Bcl-2 is a well-known and attractive therapeutic target for cancer. In the present study the solution-phase T3P-DMSO mediated efficient synthesis of 2-amino-chromene-3-carbonitriles from alcohols, malanonitrile and phenols is reported. These novel 2-amino-chromene-3-carbonitriles showed cytotoxicity in human acute myeloid leukemia (AML) cell lines. Compound **4g** was found to be the most bioactive, decreasing growth and increasing apoptosis of AML cells. Moreover, compound **4g** (at a concentration of 5 µM) increased the G2/M and sub-G1 (apoptosis) phases of AML cells. The AML cells treated with compound **4g** exhibited decreased levels of Bcl-2 and increased levels of caspase-9. *In silico* molecular interaction analysis showed that compound **4g** shared a similar global binding motif with navitoclax (another small molecule that binds Bcl-2), however compound **4g** occupies a smaller volume within the P2 hot spot of Bcl-2. The intermolecular π-stacking interaction, direct electrostatic interactions, and docking energy predicted for **4g** in complex with Bcl-2 suggest a strong affinity of the complex, rendering **4g** as a promising Bcl-2 inhibitor for evaluation as a new anticancer agent.

## Introduction

Programmed cell death, or apoptosis, is the primary mechanism for the removal of aged and damaged cells. Cancer cells can gain a growth advantage over their normal counterpart by either dividing more quickly, not undergoing terminal differentiation and thus remaining in the proliferative pool, or not undergoing apoptosis [1]. On the functional level, interactions between pro-apoptotic proteins such as Bax, Bak, Bad, Bim, Noxa, Puma, and pro-survival proteins such as Bcl-2, Bcl-xL, Bcl-w, Mcl-1, and Bfl-1 control the regulation of programmed cell death. Cancer cells alter the balance among these opposing factions to undermine normal apoptosis, and thus gain a survival advantage [2], [3]. The first identified apoptotic regulator, Bcl-2, was cloned from human follicular B cell lymphoma cells which nearly invariably have a chromosomal t(14;18) translocation, placing the Bcl-2 gene under the control of the powerful IgG heavy chain promoter [4], [5] with the consequence of elevated levels of Bcl-2 promoting increased cell survival [6]. A common feature in many human tumors is overexpression of the pro-survival Bcl-2 family members Bcl-2 and Bcl-xL, which make tumor cells resistant to conventional cancer therapeutic agents.

**Figure 1:**
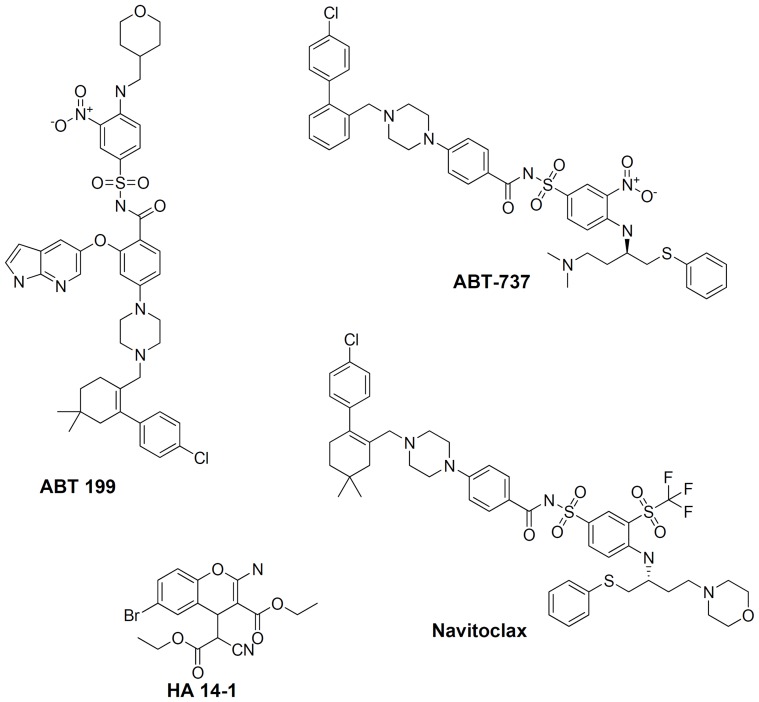
Known small molecules that target Bcl-2. As can be seen, different bioactive scaffolds have been established, however both efficacy and avoiding off-target effects of this class of compounds still remains a challenge.

Numerous synthetic small molecules targeting Bcl-2 protein have been studied extensively and few of them have advanced to clinical trials (**Figure 1**). Structure-based drug design approaches have previously yielded small molecules that bind to Bcl-2 such as navitoclax (ABT-263) [7]. This molecule binds to Bcl-2 and Bcl-xL; unfortunately in clinical trials it caused severe thrombocytopenia due to binding and inhibiting Bcl-xL [8]. Another structure-based synthesis has produced BM-957, a potent small-molecule inhibitor of Bcl-2 and Bcl-xL, which was capable of achieving complete tumor regression in a small lung cancer xenograft model [9]. Similarly, the co-crystal structure of Bcl-2 resulted in identification of a small molecule called ABT-199; a Bcl-2–selective inhibitor approved by the FDA for cancer therapy [10]. The above study strongly suggested that an indole based carbinol inhibited the growth of prostate cancer cells by arresting them in the G1 phase of the cell cycle, leading to apoptosis *via* down-regulation of Bcl-2.

Chromene-based natural and synthetic compounds have contributed substantially to the development of therapeutics as anti-neoplastic agents against various human malignancies [11], [12]. Sesilin, tephrosin, calanone and acronycine are some of the naturally occurring chromene derivatives with a very good anti-cancer activity. An important breakthrough in the development of 4*H*-chromenes as anti-cancer agents was given by the discovery of HA 14-1, which targets the Bcl-2 protein. It is reported to inhibit Bcl-2 by abrogating the interaction of Bax/Bcl-2, and it has also shown synergistic effect when combined with flavopiridol [13]–[15]. Therefore, the design of new chromene derivatives against anti-apoptotic members of the Bcl-2 family provides a plausible target in cancer therapeutics [16]. We recently reported the synthesis and explored the biological property of chromene-containing benzoxazines and other biologically important heterocyclic libraries [17], [18].

**Figure 2:**
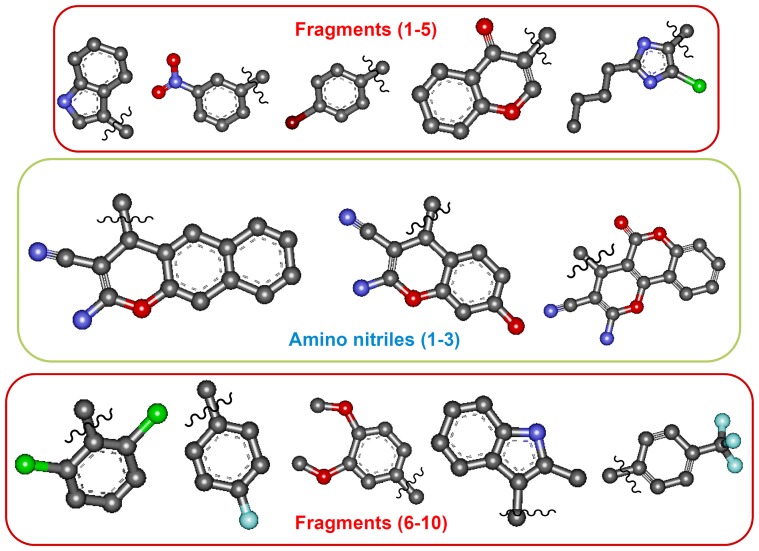
Molecular diversity of amino-nitrils. Structural representation (ball and stick model) of the combinatorial libraries of Bcl-2 inhibitors that depicts top and bottom row side chains, which are incorporate to three amino nitrile scaffolds.

In order to improve the efficacy of chromene-based small molecules as selective inhibitors of Bcl-2, we have in the current work synthesized a library of 2-amino chromene-3-carbonitriles to quantify and compare the molecular diversity between different types of libraries that would interact with Bcl-2 (**Figure 2**). We also outline the simple graphical approach for describing and comparing molecular diversity within the library of amino-nitriles.

## Materials and Methods

Synthesis and characterization of various 2-amino-chromene-nitriles were provided as supplementary data (**Data S1** and **Table S1**)

### Cell Culture

Myeloid leukemia cell lines; MOLM13 and MV4-11 were kind gifts by Dr. Martin Grundy (Department of Academic Haematology, University of Nottingham) [28]. MOLM14 cells were generously provided by Dr. Didier Bouscary (Department of Hematology-Immunology, Institut Cochin, Paris, France) [29]. The HL-60 cell line was purchased from ATCC. Cell lines were cultured and maintained in RPMI medium containing 10% fetal bovine serum (FBS), 1% penicillin-streptomycin (Invitrogen, Carlsbad, CA) at 37°C in a humidified atmosphere with 5% CO_2_. All cell lines were regularly screened for absence of mycoplasma. Bone marrow cells from C57BL/6 mice were cultured in IMDM supplemented with 20% FBS and cytokines (10 ng/ml recombinant mouse IL-3, 10 ng/ml recombinant mouse IL-6 and 50 ng/ml recombinant mouse Stem Cell Factor (SCF). Antibodies against Bcl-2, Bcl-xL, Cleaved caspase-9 and GAPDH were purchased from Cell Signalling. ABT-737 was used as a reference compound for all the experiments.

### Cell proliferation assay

Cellular proliferation of AML cell lines was measured by using methylthiazolyl-diphenyl-tetrazolium bromide (MTT; sigma-aldrich). 8,000 cells per well were seeded in triplicate, 96-well plates (Corning, Lowell, MA, USA) in 100 µl of medium having 2-amino-chromene-nitriles derivatives. Cells were incubated overnight at 37°C in a CO_2_ incubator. At the end of the culture duration, 3-(4, 5-dimethylthiazol-2-yl)-2, 5-diphenyltetrazolium bromide (MTT) was added into each well, and the final concentration of MTT in each well was 0.5 mg/ml. MTT plates were incubated at 37°C in a CO_2_ incubator for 3 hr. After incubation, Formazan crystals were dissolved in 100 µl of stop solution (SDS-HCl). Absorbance was measured at 570 nm using a Tecan Infinite 200 PRO spectrophotometer (Mannedorf, Switzerland).

### Annexin V and propidium iodide (Annexin V–PI) apoptosis assays

HL-60, MOLM13, MOLM14 and MV4-11 (1×10^5^) cells were seeded into each well of a 6-well plate and treated with **4g** at the IC_50_ concentration for 48 and 72 hours. Staining was performed using FITC Annexin V Apoptosis Detection Kit II, BD Pharmingen (BD Biosciences, USA) according to the manufacturer protocol. Briefly, cells were washed with PBS at least three times, re-suspended in 1× binding buffer, and FITC-conjugated annexin V and propidium iodide (PI) was added for 15 min in the dark. Samples were analyzed by a LSR-II flow cytometer (Becton-Dickinson, San Jose, CA, USA).

### Immunoblot analysis

Cell lysates were prepared using the ProteoJET Mammalian Cell lysis reagent (Fermentas) and the 1× protease inhibitor mixture (Roche Molecular Biochemicals, Pleasanton, CA). Immunoblotting was performed as described earlier [30]. Monoclonal antibodies against Bcl-2, GAPDH and cleaved caspase-9 (Cell Signalling, Boston, MA, USA) were used.

### *In Vitro* analysis of the effect of amino-nitriles against Bcl-2

Zymed Bcl-2 ELISA kit was used for the evaluation of the binding of small molecules to Bcl-2. Initially various concentrations of small molecules and the human Bcl-2 was incubated for 5 minutes and transferred the mixture to the mAb coated 96-well plate. The bound Bcl-2 was tagged with anti-Bcl-2 that conjugated with biotin. The biotin conjugate was bound with streptauvidin-HRP. The Streptavidin-HRP was reacted with TM and the absorbance is measured at 450 nm. A standard curve is prepared to determine the Bcl-2 concentration and% inhibition of the Bcl-2 binding to its antibody was presented.

### Molecular docking analysis

The molecular modeling was achieved with commercially available InsightII, Discovery Studio (DS) Version 2.5 software packages. Initially, the 3D structure of Bcl-2 was cleaned and the navitoclax binding site was considered for further analysis. All of the calculations were performed using the CHARMM force field as reported previously [31]. Each energy-minimized final docking position of the individual apigenin structural analogues was evaluated using the interaction score function in the CDOCKER module of DS version 2.5 as reported previously from our group [32]. Based on the low CDOCKER energy value, compound **4g** was selected for further studies.

## Results and Discussion

### Synthesis of novel 2-amino chromene-3-carbonitriles derivatives

**Figure 3:**
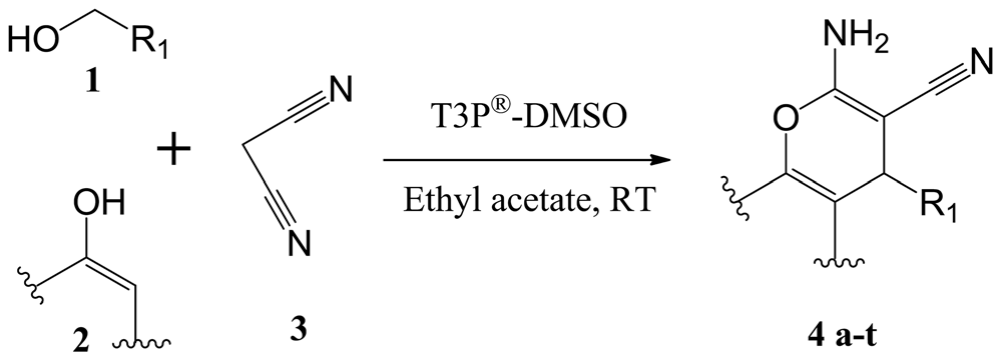
Synthesis of a library of 2-amino-chromene-3-carbonitriles from alcohols.

**Figure 4:**
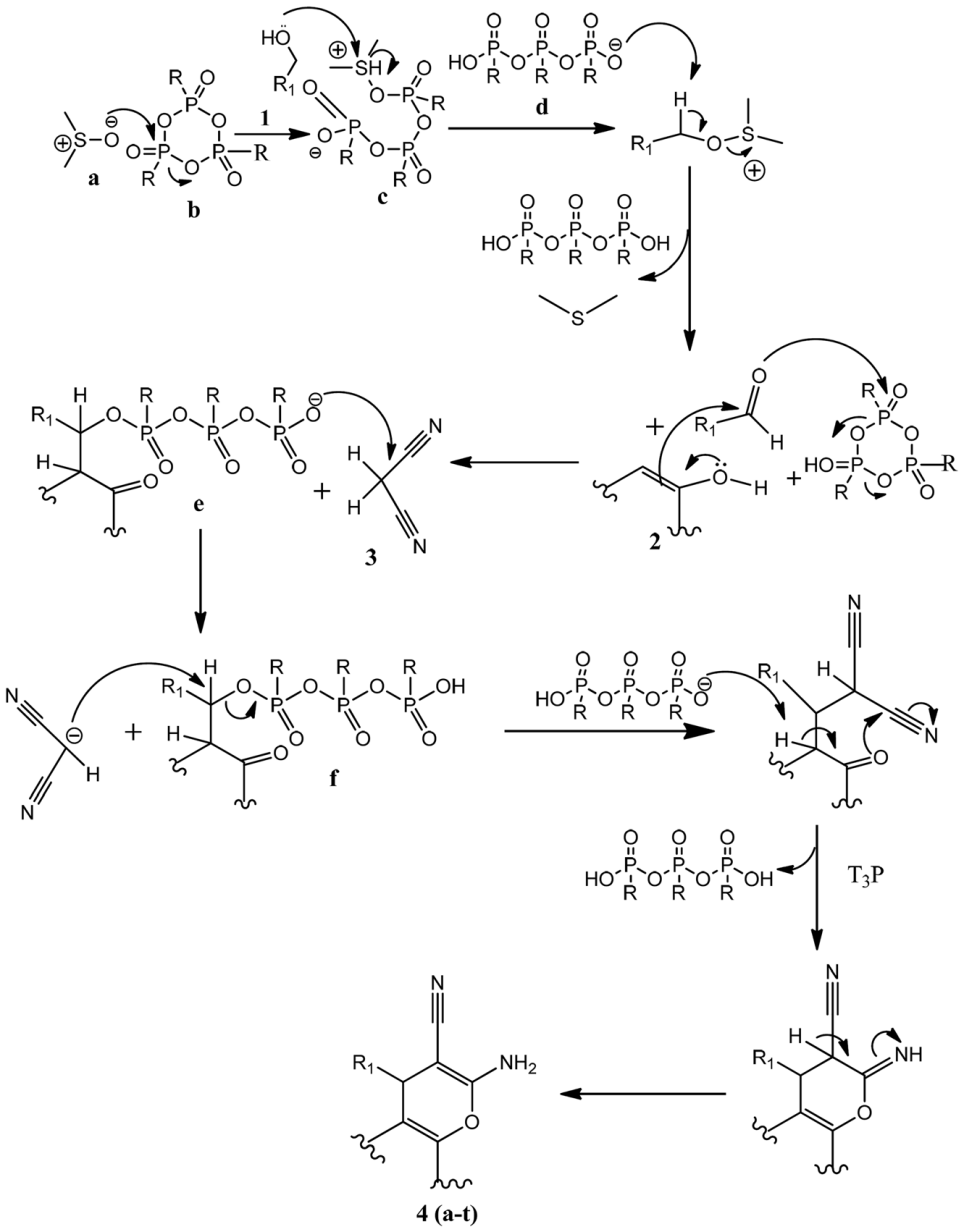
Plausible mechanism of the T3P-DMSO mediated synthesis of title compounds.

Naturally occurring 2-amino-carbonitriles containing pyrano[3,2-c]pyridine-based structural motifs are commonly found in alkaloids and exhibit diverse biological activities, including anti-cancer activity [19]. A series of pyrano[3,2-c]pyridine based flavone and coumarin isosteres containing a 2-amino carbonitrile moiety have also exhibited a broad spectrum of antitumor activity against breast cancer cells [20]. In continuation of our efforts to develop efficient solution phase synthesis of novel small molecules with anti-cancer agents, we reported before the synthesis of 2-amino-chromene-3-carbonitriles [21], [22], where solution phase T3P-DMSO mediated the efficient synthesis using alcohols, malanonitrile and phenols (**Figure 3**). The possible mechanism involves the reaction of DMSO with T3P to give an electrophilic sulphur species (c), followed by a substitution reaction of alcohol to form an arylsulphonium salt. This is followed by a hydrogen abstraction of the arylsulphonium salt by hydrolyzed T3P (d) to create a carbonyl compound after elimination of dimethyl sulfide. Next, the phenolic hydroxyl group (2) attacks the carbonyl carbon, and the oxygen on the carbonyl group attacks one more T3P to form an intermediate (e), which in turn attacks malanonitrile (3) to give an anion. This anion attacks intermediate (f), which upon further cyclization converts to compound 4(a-t) (**Figure 4**). In addition, a variety of aromatic and heterocyclic alcohols, β-naphthol/resorcinol/4-hydroxycoumarin and malanonitrile were added to T3P (50% solution in ethylacetate), DMSO and ethyl acetate as a solvent medium, in order to obtain compounds **4a-t**. The reaction between **1**, **2** and **3** in the presence of T3P (2.5 equiv) DMSO generally resulted in a product of appreciable yield (see **Table 1**) as well as being rather versatile with regard to the choice of substrates. It was observed during the reaction that an increase in temperature resulted in the gradual decrease in yield of the product.

**Table 1:** Physical characteristics of the synthesized 2-amino-chromene-3-carbonitriles.

Entry	1 (Alcohol)	3 (Phenols)	Title compounds (4a-t)	Time (Hr)	Yield (%)	MP (^O^C)
4a	(3-nitrophenyl)methanol	naphthalen-2-ol	2-amino-4-(3-nitrophenyl)-4H-benzo[g]chromene-3-carbonitrile	3–4	95^a^	179 –182
4b	(4-bromophenyl)methanol	naphthalen-2-ol	2-amino-4-(4-bromophenyl)-4H-benzo[g]chromene-3-carbonitrile	4.5	96^a^	138–140
4c	(1H-indol-3-yl)methanol	naphthalen-2-ol	2-amino-4-(1H-indol-3-yl)-4H-benzo[g]chromene-3-carbonitrile	5	92^a^	198–200
4d	3-(hydroxymethyl)-4H-chromen-4-one	naphthalen-2-ol	2-amino-4-(4-oxo-4H-chromen-3-yl)-4H-benzo[g]chromene-3-carbonitrile	9	84	-
4e	(2-butyl-4-chloro-1H-imidazol-5-yl)methanol	naphthalen-2-ol	2-amino-4-(2-butyl-4-chloro-1H-imidazol-5-yl)-4H-benzo[g]chromene-3-carbonitrile	12	79	-
4f	(2-nitrophenyl)methanol	naphthalen-2-ol	2-amino-4-(2-nitrophenyl)-4H-benzo[g]chromene-3-carbonitrile	4-5	89^a^	-
4g	(2,6-dichlorophenyl)methanol	naphthalen-2-ol	2-amino-4-(2,6-dichlorophenyl)-4H-benzo[g]chromene-3-carbonitrile	8	91	-
4h	(4-fluorophenyl)methanol	naphthalen-2-ol	2-amino-4-(4-fluorophenyl)-4H-benzo[g]chromene-3-carbonitrile	4.5	93^a^	187–189
4i	(4-fluorophenyl)methanol	resorcinol	2-amino-4-(4-fluorophenyl)-7-hydroxy-4H-chromene-3-carbonitrile	6	96^a^	218–220
4j	(1H-indol-3-yl)methanol	resorcinol	2-amino-7-hydroxy-4-(1H-indol-3-yl)-4H-chromene-3-carbonitrile	5	92^a^	-
4k	3-(hydroxymethyl)-4H-chromen-4-one	resorcinol	2′-amino-7′-hydroxy-4-oxo-4H,4′H-[3,4′-bichromene]-3′-carbonitrile	9.5	82	-
4l	(2-butyl-4-chloro-1H-imidazol-5-yl)methanol	resorcinol	2-amino-4-(2-butyl-4-chloro-1H-imidazol-5-yl)-7-hydroxy-4H-chromene-3-carbonitrile	14	74	-
4m	(4-bromophenyl)methanol	4-hydroxy-2H-chromen-2-one	2-amino-4-(4-bromophenyl)-5-oxo-4,5-dihydropyrano[3,2-c]chromene-3-carbonitrile	3.5	97^a^	254–256
4n	(1H-indol-3-yl)methanol	4-hydroxy-2H-chromen-2-one	2-amino-4-(1H-indol-3-yl)-5-oxo-4,5-dihydropyrano[3,2-c]chromene-3-carbonitrile	5.5	86^a^	215–217
4o	3-(hydroxymethyl)-4H-chromen-4-one	4-hydroxy-2H-chromen-2-one	2-amino-5-oxo-4-(4-oxo-4H-chromen-3-yl)-4,5-dihydropyrano[3,2-c]chromene-3-carbonitrile	10	72	-
4p	(2-butyl-4-chloro-1H-imidazol-5-yl)methanol	4-hydroxy-2H-chromen-2-one	2-amino-4-(2-butyl-4-chloro-1H-imidazol-5-yl)-5-oxo-4,5-dihydropyrano[3,2-c]chromene-3-carbonitrile	9	81	-
4q	(3,4-dimethoxyphenyl)methanol	4-hydroxy-2H-chromen-2-one	2-amino-4-(3,4-dimethoxyphenyl)-5-oxo-4,5-dihydropyrano[3,2-c]chromene-3-carbonitrile	2.5	82^a^	228–230
4r	(2-methyl-1H-indol-3-yl)methanol	4-hydroxy-2H-chromen-2-one	2-amino-4-(2-methyl-1H-indol-3-yl)-5-oxo-4,5-dihydropyrano[3,2-c]chromene-3-carbonitrile	6	69	-
4s	(3,4-dimethoxyphenyl)methanol	4-hydroxy-6,7-dimethyl-2H-chromen-2-one	2-amino-4-(3,4-dimethoxyphenyl)-8,9-dimethyl-5-oxo-4,5-dihydropyrano[3,2-c]chromene-3-carbonitrile	3.5	84	-
4t	(4-(trifluoromethyl)phenyl)methanol	4-hydroxy-6,7-dimethyl-2H-chromen-2-one	2-amino-8,9-dimethyl-5-oxo-4-(4-(trifluoromethyl)phenyl)-4,5-dihydropyrano[3,2-c]chromene-3-carbonitrile	4	89	-

a Isolated yield.

MP- Melting point.

### 2-amino chromene-3-carbonitrile derivatives elicit an anti-proliferative effect against AML cells

**Figure 5:**
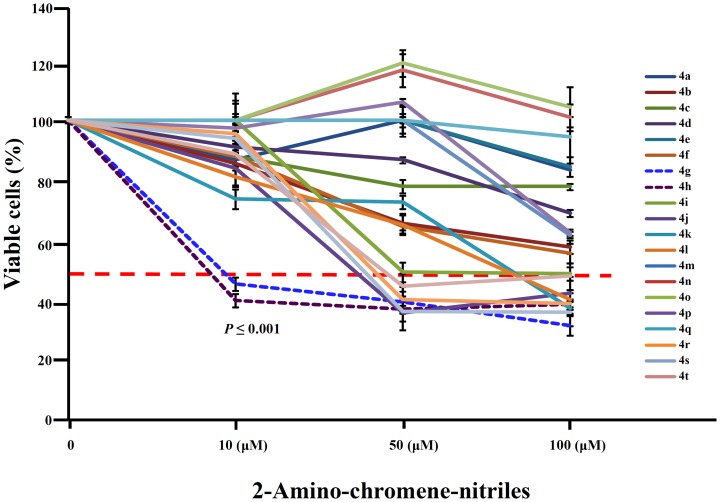
Screening of active compounds affecting the proliferation of HL-60 AML cells from a library of 2-amino-chromene-nitriles derivatives. MTT assays were performed after incubation of HL-60 cells with indicated concentrations of chromene derivatives 4 (a-y) for 72 hr. For each concentration, percent inhibition values were calculated and data was normalized to diluent controls. The scale X-axis is non-linear and the data represent mean ±SD from three independent experiments done in quadruplicates. ** *P*≤0.001 (Student's t-test).

4*H*-chromenes are potent cytotoxic agents which have been tested against a panel of human cancer cell lines, and a chromene analog, Crolibulin (EPC2407) is currently in Phase I/II clinical trials for the treatment of advanced solid tumors [23]. We initially tested a series of 20 chromene derivatives 4(a-t) for their cytotoxic effects against a human acute myeloid leukemia (AML) cell line (HL-60), using three different concentrations (10, 50 and 100 µM) of 2-amino-chromene-carbonitriles. Among the tested compounds, **4g** significantly (***P*** **≤0.001**) decreased proliferation of the AML cells, even at 10 µM concentrations. Other derivatives of these compounds showed either no or very low effect at 10 µM concentrations (**Figure 5**).

**Figure 6:**
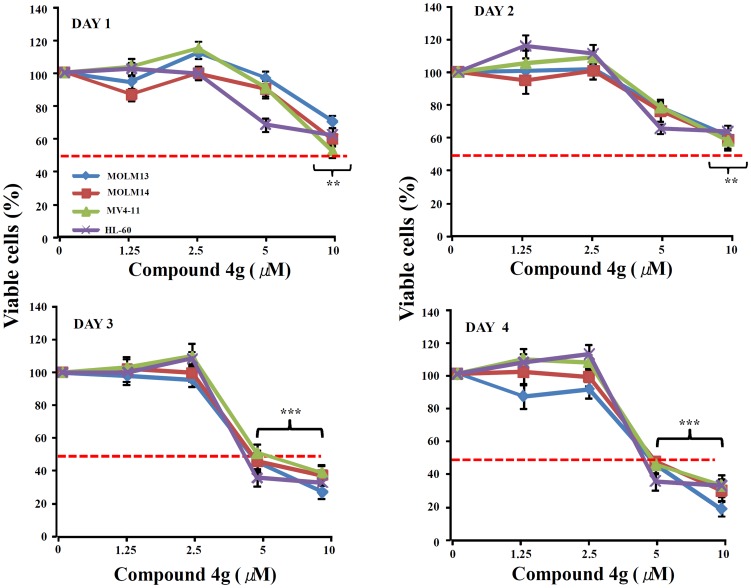
Anti-proliferative effect of 4g tested against AML cell lines in liquid culture. Panels (A–D): MTT assay determined cell viability of AML cells. 8,000 cells per well were seeded for MOLM13, MOLM14, MV4-11 and HL-60 cells in 96 well plates in quadruplicates. A series of dilutions (starting from 1.25 µm to 10 µm) of **4g** were added into the wells. Cell proliferation was measured after compound **4g** treatment relative to diluents controls. Results represent the mean ±SD of three independent experiments with quadruplicate wells per experiment point. ** *P*≤0.001; *** *P*≤0.0001 (Student's t-test).

**Figure 7:**
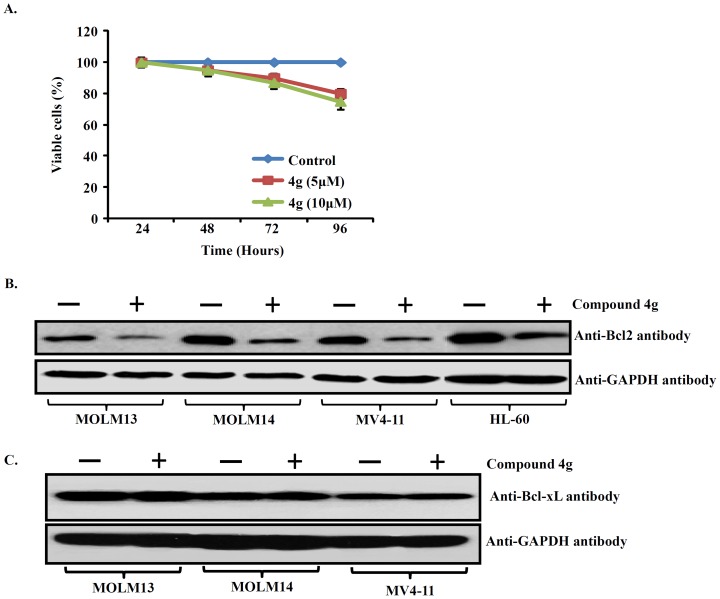
Expression of Bcl-2 proteins in human AML cell lines and anti-proliferative effect of compound 4g (5, 10 µm against C57BL/6 mouse bone marrow cells in liquid culture. **A**. MTT assay determined cell viability of C57BL/6 mouse total bone marrow cells. Results represent the mean ±SD of three independent experiments with quadruplicate wells per experiment point. **B**. MOLM13, MOLM14, MV4-11 and HL-60 AML cells were cultured either with compound **4g** (5 µM, 24 hr) or diluents control, and levels of Bcl-2 and Bcl-xL were examined by western blot. GAPDH was used as an internal loading control.

Additional studies were performed for compound **4g**, which was the most active among the structurally related compounds tested in culture. The anti-proliferative activity of compound **4g** against three additional AML cell lines (MOLM13, MOLM14 and MV4-11) was examined. Again, compound **4g** significantly decreased the proliferation of the AML cells with an ED_50_ of 5 to 10 µM after 48, 72 and 96 hours of culture (**Figures 6**). Compound **4g** inhibited proliferation of the AML cells with an IC_50_ of 5 µM. Further, C57BL/6 mouse bone marrow cells were treated with 5, 10 µM compound **4g** to assess the effect on normal hematopoietic cells. As shown in **Figure 7A**, compound **4g** (5, 10 µM) treatment displayed minimal anti-proliferative response against the normal bone cells suggesting that compound **4g** decreases proliferation of the AML cells, and has little effect on proliferation of normal bone marrow cells.

### Compound 4g inhibits Bcl-2 protein and triggers cell cycle arrest at G2/M phase in AML cells

HA14-1 is a cell permeable Bcl-2 inhibitor, and acts by binding to the surface pocket of Bcl-2 [24] and disrupts the Bax/Bcl-2 interaction, resulting in induction of apoptosis of tumour cells. Interestingly, treatment of HL-60, MOLM13, MOLM14, MV4-11 cell lines with compound **4g** (5 µM, 24 hr) decreased levels of Bcl-2 (**Figure 7B**). Moreover, we checked the effect of compound 4g on the expression of the Bcl-xL. Our western blotting results showed that compound **4g** does not inhibit the expression of Bcl-xL protein (**Figure 7C**).

**Figure 8:**
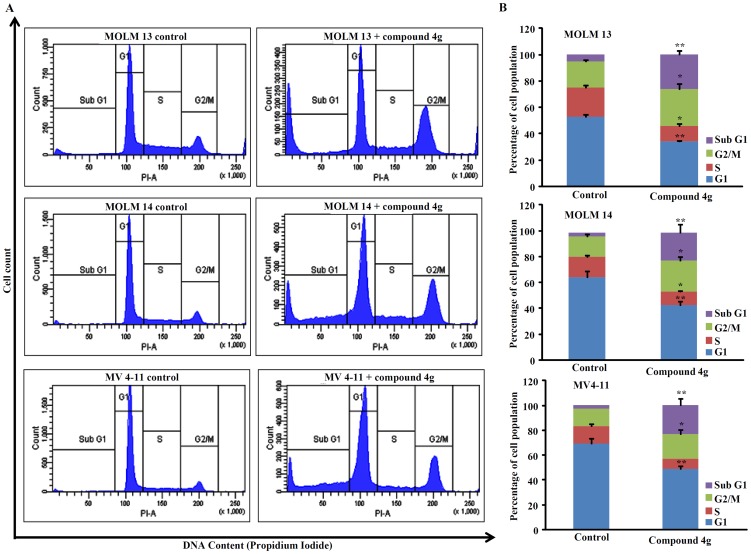
Cell cycle analysis of AML cell lines treated with compound 4g (5 µM, 72 hr). Panel (A), fluorescent activated cell sorter analyzed the percent cells in each phase of the cell cycle. Cell cycle analysis of three cell lines (MOLM14, MOLM14 and MV4-11) treated with diluents control and compound **4g** (5 µM) for 72 h. The figures are representative of three independent experiments. Data are presented in histograms as mean ±SD of three independent experiments. * *P*≤0.005; ** *P*≤0.001.

The effect of compound **4g** (5 µM, 72 hr) on the cell cycle analysis of MOLM13, MOLM14, and MV4-11 AML cells using flow cytometry showed significant increase in the G2/M and sub-G1 phases (apoptosis), as compared to the diluents control cells (**Figures 8A and 8B**). The cell population in the G1 phase of the cell cycle decreased.

### Compound 4g induces apoptosis of AML cells

**Figure 9:**
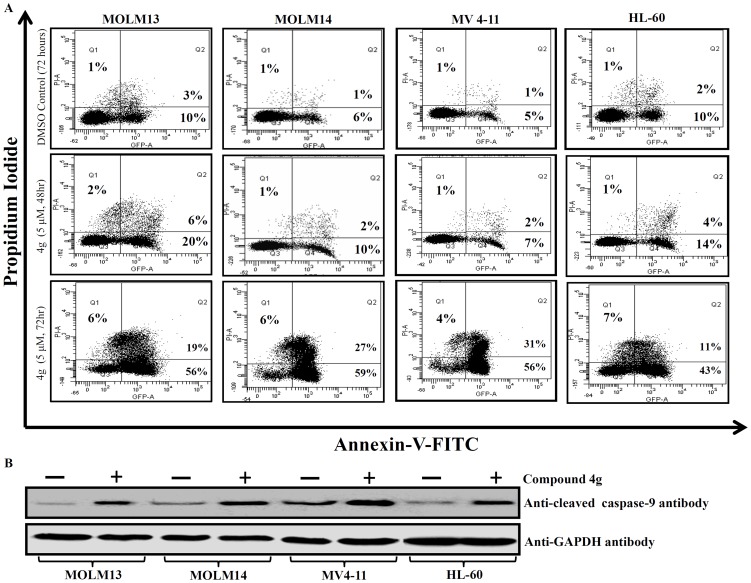
Compound 4g induces apoptosis of AML cells in a time-dependent manner. **A**. Flow cytometry profile represents Annexin V-FITC staining on the X-axis and PI staining on the Y-axis. The upper left quadrants display the necrotic cells, upper right quadrants show the late apoptotic cells, lower left quadrants display the live cells and lower right quadrants show the early apoptotic cells. **B**. Western blot showed increased expression of cleaved caspase-9 protein in compound 4g treated AML cells compared to control cells.

Core scaffolds such as flavones or chromenes induce apoptosis in human myeloid tumor cells [25]. Therefore, the HL-60, MOLM13, MOLM14, and MV4-11 cells were treated with compound **4g** (5 µM for 48 and 72 hr) and analysed for the induction of apoptosis using FITC-conjugated annexin V (AV) and propidium iodide (PI). Both early (AV^+^PI^−^), as well as, late (AV^+^PI^+^) apoptosis occurred in AML cells (**Figure 9A**). Detailed percentage of apoptotic cells in cell lines are summarized in supplementary data (**Table S1**). This was associated with increased levels of cleaved caspase-9 in these cell lines (**Figure 9B**), which is an enzyme known to be activated during programmed cell death.

### *In Vitro* analysis of the effect of amino-nitriles against Bcl-2

**Figure 10:**
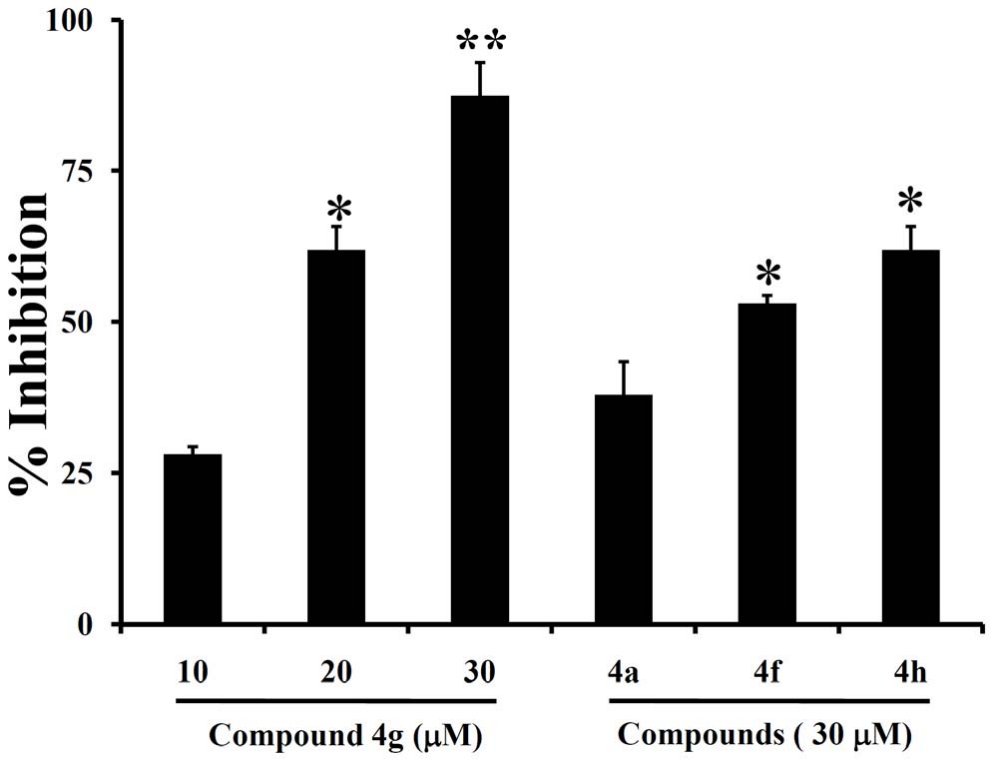
Amino-nitriles inhibit Bcl-2 *in vitro.* Most active small molecules 4a, 4f, 4g and 4h were analysed for their Bcl-2 inhibitory activity. **4g** displayed higher percentage of Bcl-2 inhibition in a dose dependent manner and presented as the most potent inhibitor of Bcl-2.

Bcl-2 ELISA kit from Zymed was used to determine the inhibitory activity of most active of small molecule towards Bcl-2. Among the four tested compounds, **4a**, **4h**, and **4f** at 30 µM concentration, inhibited the Bcl-2 binding to its antibody by 37.8, 53.0, 61.9% respectively (**Figure 10**). The most active compound **4g** inhibited the Bcl-2 binding to its antibody effectively with an IC_50_ value of 17.8 µM, indicate its strong affinity towards Bcl-2 compared to structurally related chromenes. Among the tested small molecules, 2,6- dichlorophenyl, 2-nitrophenyl, 4-fluorophenyl and 3-nitrophenyl side chain containing amino-nitrils displayed significant binding affinity towards Bcl-2 indicating that electron withdrawing group favors the effective binding to Bcl-2.

### *In silico* docking analysis of chromene based small molecule (4g) that targets Bcl-2

**Figure 11:**
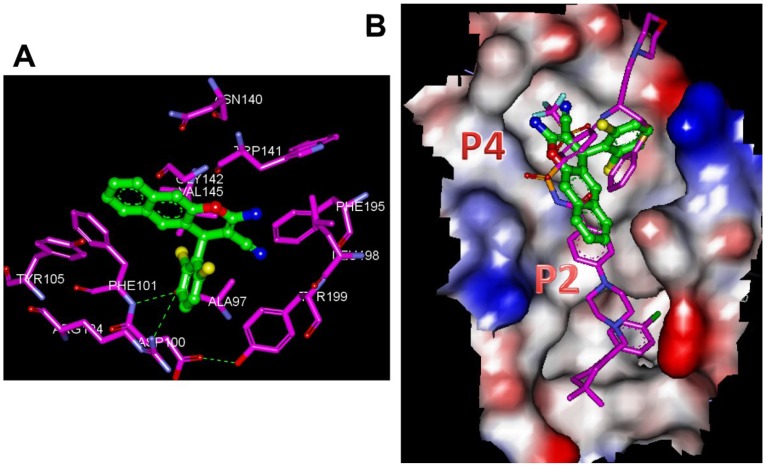
Molecular docking analysis of the compound 4g in the navitoclax binding site of Bcl-2. **A**. The 3-dimensional orientation of compound **4g** in the navitoclax binding site of Bcl-2. Amino acid side chains are shown as stick (elements are purple color except for carbon-pink), the inhibitor is shown as a ball and stick (elements are green color except for carbon-green). The hydrogen bonding is represented as a green dotted line. **B**. Navitoclax and compound 4g bound surface view of the Bcl-2, showing the interaction at the P2 and P4 hotspot of the protein. The electrostatic potential of the key amino acids is shown. The bound ball and stick version of the compound **4g** and navitoclax are represented.

Bcl-2 appears to be a possible target for compound **4g** as it induced the apoptosis by decreasing the level of anti-apoptotic protein Bcl-2 in AML cell lines. Hence, we performed *in silico* molecular docking using the three-dimensional structures of Bcl-2, which consists of two central primarily hydrophobic α-helices surrounded by amphipathic helices. Our study was aided by the recent definition of the X-ray crystal structure of Bcl-2–navitoclax (PDB ID: 4LVT) [10]. Navitoclax engages the two hydrophobic ‘hot spots’ regions termed P2 and P4 that are known to show a high-affinity binding by proapoptotic peptides [26], [27]. All docking calculations were performed using Accelrys Discovery Studio Version 2.5 and the automated docking program CDOCKER, which utilises a molecular dynamics-based simulated annealing algorithm. The active site of the enzyme was defined, and CDOCKER docked the most active compound against the navitoclax binding hotspot of Bcl-2. **Figure 10** shows the best-docked conformation of compound **4g** and navitoclox in Bcl-2 obtained from the CDOCKER program. Docking energies of the best configuration of compound **4g** and navitoclax against Bcl-2 were 4.55 and 5.45 kcal/mol, respectively. In addition, we also carried out the molecular docking analysis for the other bioactive amino nitriles with Bcl-2 and its CDOCKER energies are provided in the supplementary data (**Table S2**). The 3-dimensional illustrations of the interaction map of Bcl-2 with compound **4g**, and navitoclax, were produced using the Accelrys visualization tool (**Figure 11A**), and an intermolecular π-stacking interaction of napthyl group with Phe101 and Tyr105 of P4 host spot of Bcl-2 was observed. The 2,6-dichlorophenyl group of compound **4g** showed electrostatic interactions with the conserved arginine (Arg104) and in turn made a unique contact with the intra-molecular hydrogen bond formed by Ala97, Asp100, Phe101, and Tyr199 (**Figure 11A**). In addition to this, the amino-chromene scaffold was buried in the hydrophobic pocket of Gly142, Val145, Trp141, Asn140, Phe195, Leu198, and Tyr199. The analysis of compound **4g** bound to the novitoclox binding site showed that it shares a similar global binding motif with navitoclax, but occupies a smaller volume within the P2 hot spot (**Figure 11B**).

## Conclusions

We report for the first time the solution phase, T3P-DMSO mediated, efficient synthesis of 2-amino-chromene-3-carbonitriles from alcohols, malanonitrile and phenols. These 2-amino-chromene-3-carbonitriles were tested for their cytotoxicity using human AML cell lines. Compound **4g** was the most bioactive among the structurally related compounds. It decreased proliferation, increased caspase-9 mediated apoptosis, and caused G2/M arrest of the MOLM13, MOLM14, and MV4-11 AML cells. Our docking analysis of **4g** suggests Bcl-2 as a reasonable target, hence rendering compound **4g** as a promising Bcl-2 inhibitor for future studies as a new anticancer agent.

## Supporting Information

Table S1 **Quantification of early apoptotic and late apoptotic cells (%).** AML cells were cultured with 4g (5 µM) for either 48 or 72 hours and propidium iodide (PI) and FITC conjugated Annexin V positive cells were enumerated. Results are a representative experiment. The experiments were done three times in triplicates.
(DOCX)

Table S2 **Computational analysis of the binding of amino nitriles against Bcl-2.**
(DOCX)

Data S1 **General procedure for one pot synthesis of 2-amino chromene-3-carbonitriles.**
(DOCX)
