# Supplementary material for: Synthesis and Characterization of Novel 2-Amino-Chromene-Nitriles that Target Bcl-2 in Acute Myeloid Leukemia Cell Lines
Source: PLoS One. 2014 Sep 30;9(9):e107118. doi: 10.1371/journal.pone.0107118 (PMC4182326; doi:10.1371/journal.pone.0107118)
Supplement: Table S1 — Quantification of early apoptotic and late apoptotic cells (%). AML cells were cultured with 4g (5 µM) for either 48 or 72 hours and propidium iodide (PI) and FITC conjugated Annexin V positive cells were enumerated. Results are a representative experiment. The experiments were done three times in triplicates. (DOCX) [file pone.0107118.s001.docx]

**Synthesis and Characterization of Novel 2-Amino-Chromene-Nitriles that Target Bcl-2 in Acute Myeloid Leukemia Cell lines**

Hosadurga K. Keerthy, Manoj Garg, Chakrabhavi D. Mohan, Vikas Madan, Deepika Kanojia, Rangappa Shobith , Shivananju Nanjundaswamy, Daniel J. Mason, Andreas Bender, Basappa, Kanchugarakoppal S. Rangappa, H. Phillip Koeffler

**Table S1.**

| **Sample** | **Early apoptotic cells**  **(%)** | **Late apoptotic cells**  **(%)** |
| --- | --- | --- |
| **MOLM13 Control**  **MOLM 13 + 4g (48hr**)  **MOLM 13 + 4g (72hr**) | **10 ± 0.2**  **20 ± 3**  **56 ± 5** | **3 ± 0.1**  **6 ± 0.5**  **19 ± 3** |
| **MOLM14 Control**  **MOLM 14 + 4g (48hr**)  **MOLM 14 + 4g (72hr**) | **6 ± 0.3**  **10 ± 2**  **59 ± 6** | **1 ± 0.2**  **2 ± 0.2**  **27 ± 3** |
| **MV4-11 Control**  **MV4-11 + 4g (48hr**)  **MOLM 13 + 4g (72hr**) | **5 ± 0.1**  **7 ± 2**  **56 ± 8** | **1**  **2 ± 0.5**  **31± 5** |
| **HL-60 Control**  **HL-60 + 4g (48hr**)  **HL-60 + 4g (72hr**) | **10 ± 0.4**  **14 ± 4**  **43 ± 5** | **2 ± 0.1**  **4 ± 0.2**  **11 ± 3** |

**Table S1:** Quantification of early apoptotic and late apoptotic cells (%). AML cells were cultured with 4g (5 μM) for either 48 or 72 hours and propidium iodide (PI) and FITC conjugated Annexin V positive cells were enumerated. Results are a representative experiment. The experiments were done three times in triplicates.
