# Supplementary material for: Synthesis and Characterization of Novel 2-Amino-Chromene-Nitriles that Target Bcl-2 in Acute Myeloid Leukemia Cell Lines
Source: PLoS One. 2014 Sep 30;9(9):e107118. doi: 10.1371/journal.pone.0107118 (PMC4182326; doi:10.1371/journal.pone.0107118)
Supplement: Table S2 — Computational analysis of the binding of amino nitriles against Bcl-2. (DOCX) [file pone.0107118.s002.docx]

**Synthesis and Characterization of Novel 2-Amino-Chromene-Nitriles that Target Bcl-2 in Acute Myeloid Leukemia Cell lines**

Hosadurga K. Keerthy, Manoj Garg, Chakrabhavi D. Mohan, Vikas Madan, Deepika Kanojia, Rangappa Shobith , Shivananju Nanjundaswamy, Daniel J. Mason, Andreas Bender, Basappa, Kanchugarakoppal S. Rangappa, H. Phillip Koeffler

**Table S2**

| **Compounds** | **CDOCKER ENEGRY** |
| --- | --- |
| **4a** | **-10.45 kcal/mol** |
| **4f** | **01.22 kcal/mol** |
| **4g** | **4.55 kcal/mol** |
| **4h** | **02.78 kcal/mol** |
| **navitoclax** | **5.45 kcal/mol** |

**Table S2: Computational analysis of the binding of amino nitriles against Bcl-2.**
