# Supplementary material for: Synthesis and Characterization of Novel 2-Amino-Chromene-Nitriles that Target Bcl-2 in Acute Myeloid Leukemia Cell Lines
Source: PLoS One. 2014 Sep 30;9(9):e107118. doi: 10.1371/journal.pone.0107118 (PMC4182326; doi:10.1371/journal.pone.0107118)
Supplement: Data S1 — General procedure for one pot synthesis of 2-amino chromene-3-carbonitriles. (DOCX) [file pone.0107118.s003.docx]

**Synthesis and Characterization of Novel 2-Amino-Chromene-Nitriles that Target Bcl-2 in Acute Myeloid Leukemia Cell lines**

Hosadurga K. Keerthy, Manoj Garg, Chakrabhavi D. Mohan, Vikas Madan, Deepika Kanojia, Rangappa Shobith , Shivananju Nanjundaswamy, Daniel J. Mason, Andreas Bender, Basappa, Kanchugarakoppal S. Rangappa, H. Phillip Koeffler

**Data S1**

**General procedure for one pot synthesis of 2-amino chromene-3-carbonitriles:** To a solution of primary alcohol (1.0 mmol) in a mixture of solvents Ethyl acetate: DMSO (2: 1 ratio) was added T3P^®^ (2.5 mmol, 50% solution in ethyl acetate) at RT. This was followed by the addition of malanonitrile (1.2 mmol) and β-naphthol/resorcinol/4-hydroxy coumarin (1.0 mmol). Reaction was monitored by TLC (Hexane:EtOAc 7:3). Reaction mixture was stirred stipulated time at room temperature. After completion of the reaction, the mixture was diluted with water. The product was extracted with ethyl acetate, and the combined organic layers were washed with water followed by brine solution. The organic phase was dried over anhydrous Na_2_SO_4_. The solvent was removed under reduced pressure to afford a crude product which was purified by column chromatography using hexane: ethyl acetate mixture (7:3) as an eluent. Spectral properties are consistent with the assigned structures of novel 2-amino chromene-3-carbonitriles and the reported structural analysis of molecules matched those that were synthesized in our laboratory.

**2-amino-4-(4-oxo-4H-chromene-3-yl)-4H-benzo[g]chromene-3-carbonitrile (4d):** Brown Solid, IR ν_max_: 3352 cm^-1^ ν_(NH2)_, 2189 cm^-1^ ν_(CN)_, 1736 cm^-1^ ν_(C=O)_, 1655 cm^-1^ ν_(vinyl nitrile)_ ; ^1^H NMR (DMSO, 400 MHz) δ 2.083 (2H, -NH_2_, s), δ 4.568 (1H, Methine, s), δ 6.932 (1H, Ar-H, s), δ 7.088-7.180 (2H, Ar-H, m), δ 7.192-7.291 (2H, Ar-H, m), δ 7.302-7.392 (1H, Ar-H, m), δ 7.401-7.531 (1H, Ar-H, m), δ 7.570-7.706 (2H, Ar-H, m), δ 7.772-7.832 12H, Ar-H, m); ^13^C NMR (CDCl_3_, 300MHz) δ 30.13, δ 57.95, δ 108.83, δ 115.60, δ 117.66, δ 119.48, δ 122.53, δ 123.46, δ 123.57, δ 125.17, δ 125.75, δ 127.35, δ 128.46, δ 128.95, δ 129.44, δ 131.08, δ 132.53, δ 135.57, δ 151.13, δ 155.79, δ 157.22, δ 175.57, δ 182.53; LCMS (MM:ES+APCI) (M+H)^+^ 367.

**2-amino-4-(2-butyl-4-chloro-1H-imidazol-5-yl)-4H-benzo[g]chromene-3-carbonitrile (4e):** Brown Solid, IR ν_max_: 3389 cm^-1^ ν_(NH2)_, 2152 cm^-1^ ν_(CN)_, 1671 cm^-1^ ν_(vinyl nitrile)_ , 725 cm^-1^ ν_(C-Cl)_; ^1^H NMR (DMSO, 400 MHz) δ 0.799-0.903 (3H, -CH_3_, m), δ 1.235-1.299 (2H, -CH_2_-, m), δ 1.416-1.518 (2H, -CH_2_, m), δ 2.006-2.043 (2H, -CH_2_-, m), δ 2.083 (2H, -NH_2_, s), δ 4.486 (1H, Methine, s), δ 7.138-7.237 (2H, Ar-H, m), δ 7.405-7.578 (2H, Ar-H, m), δ 7.696-7.726 (2H, Ar-H, m), δ 9.819 (1H, -NH-, s); ^13^C NMR (CDCl_3_, 300MHz) δ 14.52, δ 21.77, δ 23.04, δ 31.79, δ 33.48, δ 58.33, δ 107.32, δ 115.83, δ 123.07, δ 124.20, δ 125.14, δ 125.69, δ 126.12, δ 127.43, δ 128.62, δ 129.77, δ 131.81, δ 133.04, δ 147.95, δ 155.57, δ 177.14; LCMS (MM:ES+APCI) (M+H)^+^ 379.

**2-amino-4-(2,6-dichlorophenyl)-4H-benzo[g]chromene-3-carbonitrile (4g):** Brown Solid; IR ν_max_: 3362 cm^-1^ ν_(NH2)_, 2171 cm^-1^ ν_(CN)_, 1707 cm^-1^ ν_(vinyl nitrile)_ , 725 cm^-1^ ν_(C-Cl)_; ^1^H NMR (DMSO, 400 MHz) δ 2.109 (2H, -NH_2_, s), δ 4.900 (1H, Methine, s), δ 7.056-7.120 (1H, Ar-H, m), δ 7.201-7.273 (1H, Ar-H, m), δ 7.365-7.421 (2H, Ar-H, m), δ 7.506-7.592 (2H, Ar-H, m), δ 7.607 (1H, Ar-H, s), δ 7.674-7.782 (1H, Ar-H, m), δ 7.803-7.910 (1H, Ar-H, m); ^13^C NMR (CDCl_3_, 300 MHz) δ 13.22, δ 56.15, δ 104.56, δ 118.24, δ 123.64, δ 124.85, δ 125.34, δ 126.08, δ 127.29, δ 127.74, δ 128.05, δ 128.99, δ 129.47, δ 130.10, δ 133.05, δ 135.00, δ 135.08, δ 143.89, δ 154.49, δ 177.63; LCMS (MM:ES+APCI) (M+H)^+^ 368.

**2-amino-4-(4-oxo-4H-chromene-3-yl)-7-hydroxy-chromene-3-carbonitrile (4k):** Pale red Solid; IR ν_max_: 3313 cm^-1^ ν_(NH2)_, 2228 cm^-1^ ν_(CN)_, 1707 cm^-1^ ν_(C=O)_, 1655 cm^-1^ ν_(vinyl nitrile)_; ^1^H NMR (DMSO, 400 MHz) δ δ 2.003 (2H, -NH_2_, s), δ 4.115 (1H, Methine, s), δ 5.184 (1H, -OH, s), δ 6.968-7.034 (1H, Ar-H, m), δ 7.071-7.183 (2H, Ar-H, m), δ 7.206-7.273 (2H, Ar-H, m), δ 7.309-7.354 (1H, Ar-H, m), δ 7.380-7.462 (1H, Ar-H, s), δ 7.491-7.565 (1H, Ar-H, m); ^13^C NMR (CDCl_3_, 300 MHz) δ 35.10, δ 56.61, δ 104.77, δ 114.23, δ 125.82, δ 126.26, δ 126.93, δ 127.43, δ 128.44, δ 130.32, δ 133.09, δ 135.10, δ 138.03, δ 140.36, δ 152.22, δ 153.85, δ 155.53, δ 177.23, δ 182.36; LCMS (MM:ES+APCI) (M+H)^+^ 333.

**2-amino-4-(2-butyl-4-chloro-1H-imidazol-5-yl)-7-hydroxy-chromene-3-carbonitrile (4l):** Brown Solid; IR ν_max_: 3506 cm^-1^ ν_(OH)_, 3258 cm^-1^ ν_(NH2)_, 2180 cm^-1^ ν_(CN)_, 1646 cm^-1^ ν_(vinyl nitrile)_ , 722 cm^-1^ ν_(C-Cl)_; ^1^H NMR (DMSO, 400 MHz) δ 0.873-0.902 (3H, -CH_3_, m), δ 1.226-1.352 (2H, -CH_2_-, m), δ 1.499-1.555 (2H, -CH_2_, m), δ 2.000 (2H, -NH_2_, s), δ 2.451-2.470 (2H, -CH_2_-, m), δ 4.744 (1H, Methine, s), δ 5.672 ( 1H, -OH, s), δ 6.376 (1H, Ar-H, m), δ 6.497-6.535 (1H, Ar-H, m), δ 6.711-6.732 (1H, Ar-H, m), δ 11.876 (1H, -NH-, s); ^13^C NMR (CDCl_3_, 300MHz) δ 17.20, δ 24.94, δ 25.54, δ 31.22, δ 33.15, δ 58.81, δ 104.32, δ 106.95, δ 110.26, δ 118.50, δ 126.54, δ 130.05, δ 133.54, δ 143.56, δ 152.85, δ 153.63, δ 177.05; LCMS (MM:ES+APCI) (M+H)^+^ 345.

**2-amino-4-(4-oxo-4H-chromene-3-yl)-5-oxo-4,5-dihydropyrano [3,2-c]chromene-3-carbonitrile (4o):** Orange Solid, IR ν_max_: 2962 cm^-1^ ν_(NH2)_, 2196 cm^-1^ ν_(CN)_, 1673 cm^-1^ ν_(C=O)_ , 1606 cm^-1^ ν_(vinyl nitrile)_; ^1^H NMR DMSO, 400 MHz:- δ 3.155 (1H, Methine, s), δ 6.836 (1H, Ar-H, s), δ 7.178-7.239 (1H, Ar-H, m), δ 7.381-7.421 (1H, Ar-H, m), δ 7.452-7.456 (1H, Ar-H, m), δ 7.470-7.473 (1H, Ar-H, m), δ 7.490-7.494 (1H, Ar-H, m), δ 7.570-7.591 (1H, Ar-H, m), δ 7.713-7.718 (1H, Ar-H, m), δ 7.731-7.735 (1H, Ar-H, m); ^13^C NMR (CDCl_3_, 300MHz) δ 18.11, δ 56.22, δ 95.24, δ 114.37, δ 116.29, δ 116.51, δ 116.96, δ 120.54, δ 122.15, δ 123.42, δ 125.49, δ 127.32, δ 129.85, δ 134.21, δ 148.33, δ 150.51, δ 156.28, δ 158.77, δ 159.01, δ 160.20, δ 182.11; LCMS (MM:ES+APCI) (M+H)^+^ 385.

**2-amino-4-(2-butyl-4-chloro-1H-imidazol-5-yl)-5-oxo-4,5-dihydropyrano [3,2-c]chromene-3-carbonitrile (4p):** Pale Yellow Solid, IR ν_max_: 3228 cm^-1^ ν_(NH2)_, 2193 cm^-1^ ν_(CN)_, 1718 cm^-1^ ν_(C=O)_ , 1670 cm^-1^ ν_(vinyl nitrile)_; ^1^H NMR DMSO, 400 MHz:- δ 0.871-0.952 (3H, -CH_3_, m), δ 1.215-1.307 (2H, -CH_2_-, m), δ 1.506-1.581 (2H, -CH_2_-, m), δ 2.292-2.324 (2H, -CH_2_-, m), δ 4.570 (1H, Methine, s), δ 7.398-7.435 (1H, Ar-H, m), δ 7.447-7.523 (1H, Ar-H, m), δ 7.708-7.751 (1H, Ar-H, m), δ 7.883-7.903 (1H, -CH_2_-, m); ^13^C NMR (CDCl_3_, 300MHz) δ 15.32, δ 18.51, δ 23.45, δ 32.92, δ 34.27, δ 59.11, δ 106.33, δ 118.36, δ 118.97, δ 122.49, δ 124.08, δ 126.59, δ 127.82, δ 128.45, δ 136.14, δ 149.07, δ 151.22, δ 160.31, δ 161.25, δ 162.00; LCMS (MM:ES+APCI) (M+H)^+^ 397.

**2-amino-4-(2-methyl-1H-indol-3yl)-5-oxo-4,5-dihydropyrano [3,2-c]chromene-3-carbonitrile (4r):** Yellow Solid; IR ν_max_: 3352 cm^-1^ ν_(NH2)_, 2217 cm^-1^ ν_(CN)_, 1649 cm^-1^ ν_(C=O)_ , 1567 cm^-1^ ν_(vinyl nitrile)_; ^1^H NMR DMSO, 300 MHz:- δ 3.35 (3H, -CH_3_, s), δ 4.15 (1H, Methine, s), δ 7.21-7.27 (2H, Ar-H, m), δ 7.29 (1H, Ar-H, m), δ 7.44-7.47 (1H, Ar-H, m), δ 7.78-7.90 (1H, Ar-H, m), δ 8.01-8.09 (1H, Ar-H, m), δ 8.10-8.31 (2H, Ar-H, m); ^13^C NMR (CDCl_3_, 300MHz) δ 12.52, δ 30.67, δ 57.65, δ 108.93, δ 109.77, δ 112.26, δ 116.61, δ 117.09, δ 117.12, δ 121.89, δ 122.02, δ 123.54, δ 124.33, δ 126.07, δ 127.4, δ 130.20, δ 132.85, δ 136.71, δ 151.29, δ 152.97, δ 161.36, δ 163.52; LCMS (MM:ES+APCI) (M-H)^-^ 368.

**2-amino-4-(3,4-dimethoxyphenyl)-8,9-dimethyl-5-oxo-4,5-dihydropyrano [3,2-c]chromene-3-carbonitrile (4s):** White Solid; IR ν_max_:3323 cm^-1^ ν_(NH2)_, 2195 cm^-1^ ν_(CN)_, 1703 cm^-1^ ν_(C=O)_ , 1668 cm^-1^ ν_(vinyl nitrile)_; ^1^H NMR DMSO, 300 MHz:- δ 2.33 (6H, -CH_3_, s), δ 3.71 (6H, -CH_3_, s), δ 4.37 (1H, Methine, s), δ 6.70-6.89 (2H, Ar-H, m), δ 7.28-7.32 (2H, Ar-H, m), δ 7.64 (1H, Ar-H, m); ^13^C NMR (CDCl_3_, 300MHz) δ 18.97, δ 19.77, δ 38.66, δ 56.47, δ 56.51, δ 57.22, δ 106.21, δ 115.25, δ 115.91, δ 116.93, δ 117.38, δ 120.55, δ 123.48, δ 126.34, δ 132.42, δ 136.21, δ 137.11, δ 145.21, δ 146.11, δ 148.71, δ 158.32, δ 160.13, δ 162.27; LCMS (MM:ES+APCI) (M+H)^+^ 405.

**2-amino-4-(4-(trifluoromethyl)phenyl)-8,9-dimethyl-5-oxo--4,5-dihydropyrano [3,2-c]chromene-3-carbonitrile (4t):** White Solid; IR ν_max_: 3324 cm^-1^ ν_(NH2)_, 2215 cm^-1^ ν_(CN)_, 1707 cm^-1^ ν_(C=O)_ , 1668 cm^-1^ ν_(vinyl nitrile)_; ^1^H NMR DMSO, 300 MHz:- δ 3.34 (6H, -CH_3_, s), δ 4.37 (1H, Methine, s), δ 6.70 (1H, Ar-H, m), δ 6.72-6.73 (1H, Ar-H, m), δ 6.81 (1H, Ar-H, m), δ 7.21-7.24 (1H, Ar-H, m), δ 7.28-.7.32 (1H, Ar-H, m), δ 7.64 (1H, Ar-H, m); ^13^C NMR (CDCl_3_, 300MHz) δ 18.97, δ 19.77, δ 36.43, δ 55.46, δ 58.13, δ 103.06, δ 110.41, δ 111.45, δ 111.84, δ 116.88, δ 119.30, δ 119.53, δ 122.16, δ 133.17, δ 135.97, δ 142.76, δ 147.87, δ 148.87, δ 150.54, δ 153.27, δ 157.95, δ 159.79;LCMS (MM:ES+APCI) (M+H)^+^ 413.
